# Detection of SARS-CoV-2 B.1.351 (Beta) Variant through Wastewater Surveillance before Case Detection in a Community, Oregon, USA

**DOI:** 10.3201/eid2806.211821

**Published:** 2022-06

**Authors:** Melissa Sutton, Tyler S. Radniecki, Devrim Kaya, Dana Alegre, Matthew Geniza, Anne-Marie Girard, Katherine Carter, Mark Dasenko, Justin L. Sanders, Paul R. Cieslak, Christine Kelly, Brett M. Tyler

**Affiliations:** Oregon Health Authority, Portland, Oregon, USA (M. Sutton, P.R. Cieslak);; Oregon State University, Corvallis, Oregon (T.S. Radniecki, D. Kaya, D. Alegre, M. Geniza, A.-M. Girard, K. Carter, M. Dasenko, J.L. Sanders, C. Kelly, B.M. Tyler)

**Keywords:** COVID-19, coronavirus disease, SARS-CoV-2, severe acute respiratory syndrome coronavirus 2, viruses, respiratory infections, zoonoses, vaccine-preventable diseases, wastewater-based epidemiology, wastewater surveillance, genomic epidemiology, public health, next-generation sequencing, NGS, multilocus sequence typing, MLST, Oregon

## Abstract

Genomic surveillance has emerged as a critical monitoring tool during the SARS-CoV-2 pandemic. Wastewater surveillance has the potential to identify and track SARS-CoV-2 variants in the community, including emerging variants. We demonstrate the novel use of multilocus sequence typing to identify SARS-CoV-2 variants in wastewater. Using this technique, we observed the emergence of the B.1.351 (Beta) variant in Linn County, Oregon, USA, in wastewater 12 days before this variant was identified in individual clinical specimens. During the study period, we identified 42 B.1.351 clinical specimens that clustered into 3 phylogenetic clades. Eighteen of the 19 clinical specimens and all wastewater B.1.351 specimens from Linn County clustered into clade 1. Our results provide further evidence of the reliability of wastewater surveillance to report localized SARS-CoV-2 sequence information.

Since its emergence in late 2019, more than 481 million COVID-19 cases have been confirmed worldwide (*1*) and >79 million cases reported in the United States (*2*). Numerous variants of the causative virus, SARS-CoV-2, have emerged; variants of concern have demonstrated characteristics of public health concern, including increased transmissibility or clinical severity, reduced vaccine or therapeutic effectiveness, or diagnostic escape (*3*). SARS-CoV-2 genomic surveillance has quickly become an essential tool for tracking transmission and coordinating response (*4*,*5*). Individual-level genomic surveillance relies on the testing of infected persons, which, in turn, requires testing access and acceptance. In the United States, testing access has improved dramatically over the course of the pandemic but remains limited, particularly in disproportionately affected communities (*6*), and testing acceptance remains an obstacle to effective disease mitigation (*7*).

SARS-CoV-2 is shed in feces, and wastewater surveillance has emerged as complementary cost-effective community-level surveillance independent of testing access and acceptance or symptomatic infection (*8*–*10*). Using wastewater testing for SARS-CoV-2 genomic surveillance avoids the testing and symptomatic biases inherent to the sequencing of individual specimens; however, interpreting sequence data from complex mixtures of viruses at a population-level remains challenging (*11*). Multilocus sequence typing (MLST) is a method traditionally used to identify species or variants in environmental samples, including rivers, urban streams, hospital sewage, and wastewater treatment plant influents and effluents (*12*,*13*). MLST is well-suited for the analysis of wastewater RNA because it detects a set of mutations unique to a variant and does not require these mutations to be present on a single molecule of RNA (*12*–*18*).

During the COVID-19 pandemic, Oregon has been among the US states with the lowest cumulative case rates and among those with the highest proportion of cumulative molecular specimens sequenced in the United States (*19*). As of March 31, 2021, a total of 159,455 confirmed cases of COVID-19 had been identified in Oregon, and specimens from 5,674 (3.6%) of the cases had been sequenced and published in the GISAID database (https://www.gisaid.org) (*20*). At that time, the dominant SARS-CoV-2 variant circulating in Oregon was B.1.427/B.1.429 (Epsilon), followed by B.1.2 and B.1.1.7 (Alpha); only 25 B.1.351 (Beta) and 8 P.1 (Gamma) variants had been identified.

On April 19, 2021, Oregon mandated reporting of all SARS-CoV-2 variants of concern to public health authorities; before this, genomic surveillance relied largely on deidentified data submitted to GISAID. Statewide sequencing partners have been asked to submit all individual specimen sequencing results to the State of Oregon phylodynamics resource in GISAID (https://www.gisaid.org/phylodynamics/oregon-usa). 

In September 2020, the Oregon Health Authority launched wastewater surveillance in collaboration with the Oregon State University (OSU) Team-Based Rapid Assessment of Community-Level Coronavirus Epidemics (TRACE) project; >40 communities comprising ≈60% of Oregon’s population currently participate. Through this program, wastewater samples from the influent of all wastewater treatment facilities are collected at least weekly and sent to OSU for SARS-CoV-2 viral RNA quantification and sequencing of all positive samples with sufficient viral loads. Through this statewide SARS-CoV-2 wastewater surveillance platform, we demonstrate the use of MLST to detect the emergence of the SARS-CoV-2 B.1.351 (Beta) variant in rural Oregon in late March 2021, before its detection in reported cases, illustrating the ability of wastewater-based epidemiology to detect emerging variants of concern.

## Methods

### Wastewater RNA Extraction

Participating facilities collected wastewater composite samples from Albany(Linn County), Corvallis (Benton County), and Dallas (Polk County), Oregon, USA, during March 26–April 21, 2021, according to routine practice for the Oregon Wastewater Surveillance Program (Table). In brief, 24-hour time-weighted composite wastewater samples were collected weekly from the influent of Albany and Dallas wastewater treatment plants and from wastewater conveyance lines in Corvallis because of micro-sewershed surveillance at a local university. Samples were vacuum-filtered (10–50 mL) onsite through a 0.45-µm pore size, 47-mm diameter mixed cellulose ester electronegative filter (MF-Millipore, https://www.emdmillipore.com). Filters were placed into a 2-mL tube containing garnet (0.5 mm) beads and DNA/RNA Shield (Zymo Research, https://www.zymoresearch.com) to stabilize the RNA during the shipment to OSU for processing. 

**Table:** SARS-CoV-2 variant B.1.351 mutations detected in clinical specimens and wastewater samples in Linn County, Oregon, and surrounding jurisdictions, March-May 2021

Sample*	Collection site†	Date	Mutations specific to‡									
			B.1.351				Clade 1				Subclade§	
			+	–	?		+	–	?		1a	1b
Clinical specimens												
EPI_ISL_1866415	Linn Co.	2021 Mar 29¶	9	0	0		5	1	0		–	–
EPI_ISL_1736521	Linn Co.	2021 Apr 5	9	0	0		6	0	0		+	–
EPI_ISL_1736532	Linn Co.	2021 Apr 5	9	0	0		6	0	0		+	–
EPI_ISL_1737841	Linn Co.	2021 Apr 7	9	0	0		0^#^	0	0		–	–
EPI_ISL_1964160	Linn Co.	2021 Apr 9	8	0	1		6	0	0		–	+
EPI_ISL_1999265	Linn Co.	2021 Apr 12	9	0	0		5	1	0		–	–
EPI_ISL_2202145	Linn Co.	2021 Apr 16	8	0	1		4	0	2		+	–
EPI_ISL_2139637	Linn Co.	2021 Apr 26	9	0	0		6	0	0		+	–
EPI_ISL_2139638	Linn Co.	2021 Apr 26	9	0	0		6	0	0		+	–
EPI_ISL_2139639	Linn Co.	2021 Apr 26	9	0	0		6	0	0		+	–
EPI_ISL_2139644	Linn Co.	2021 Apr 27	9	0	0		6	0	0		+	–
EPI_ISL_2250177	Linn Co.	2021 Apr 27	8	0	1		6	0	0		–	+
EPI_ISL_2086679	Linn Co.	2021 Apr 28	9	0	0		6	0	0		+	–
EPI_ISL_2086678	Linn Co.	2021 Apr 28	9	0	0		6	0	0		+	–
EPI_ISL_2139636	Linn Co.	2021 Apr 30	9	0	0		6	0	0		+	–
EPI_ISL_2086694	Linn Co.	2021 Apr 30	9	0	0		6	0	0		+	–
EPI_ISL_2339336	Linn Co.	2021 May 10	9	0	0		6	0	0		+	–
EPI_ISL_2382524	Linn Co.	2021 May 12	9	0	0		6	0	0		+	–
EPI_ISL_2382527	Linn Co.	2021 May 14	9	0	0		6	0	0		+	–
Wastewater samples												
ALB-Inf-03-26-21-A	Albany, Linn Co.	2021 Mar 26	7	1	1		3	1	2		–	+
ALB-Inf-03-31-21-A	Albany, Linn Co.	2021 Mar 31	9	0	0		5	0	1		+	+
COR-25th-04-04-21-A	Corvallis, Benton Co.	2021 Apr 4	9	0	0		6	0	0		+	–
COR-26th-04-04-21-A	Corvallis, Benton Co.	2021 Apr 4	6	2	1		5	0	1		–	–
ALB-Inf-04-07-21-A	Albany, Linn Co.	2021 Apr 7	8	1	0		6	0	0		+	+
DAL-Inf-04-19-21-A	Dallas, Polk Co.	2021 Apr 19	9	0	0		6	0	0		–	+
ALB-Inf-04-21-21-A	Albany, Linn Co.	2021 Apr 21	5	3	1		2	3	1		–	–

**Figure 1:**
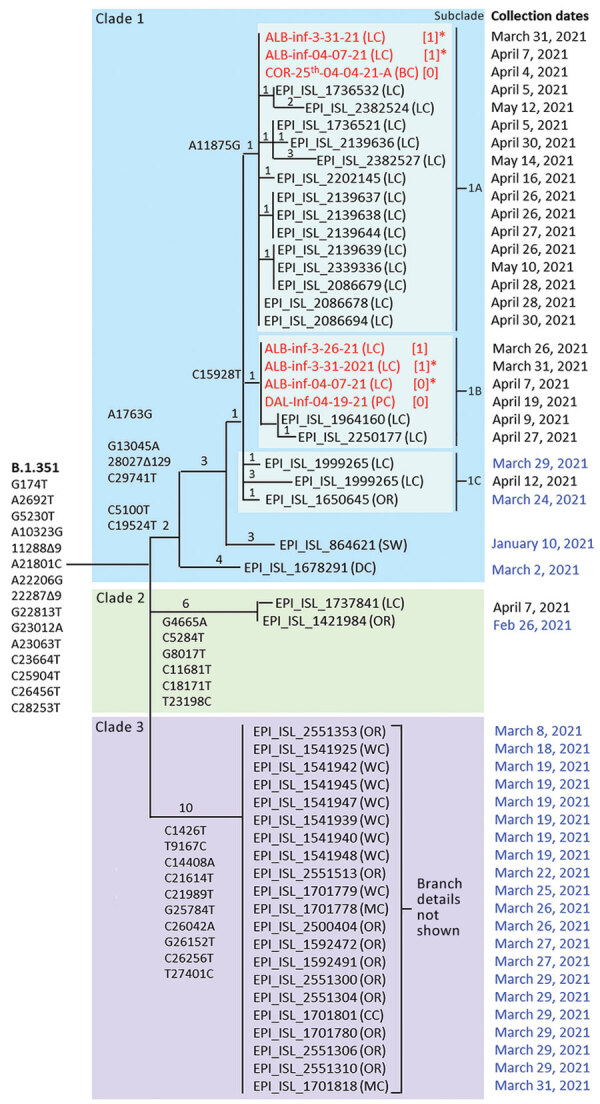
Maximum-parsimony tree demonstrating phylogenetic relationships among SARS-CoV-2 variant B.1.351 clinical specimens and wastewater samples in Linn County, Oregon, USA, and surrounding jurisdictions, March–May 2021. GISAID accession numbers (https://www.gisaid.org) are shown for 19 of 20 B.1.351 specimens identified in Linn County through May 15, 2021, and for 24 additional B.1.351 specimens identified in Oregon through March 31, 2021 (dates in blue). Also included are 2 sequences from outside Oregon (Switzerland and Washington, DC, USA) most closely related to clade 1. Wastewater samples are in red. Exact parsimony trees are shown for clade 1 and 2 sequences, whereas clade 3 sequences are simply listed. Mutations defining B.1.351 and each of the 3 clades, plus subclades 1a and 1b, are shown. Private mutations defining the subbranches of clades 1 and 2 are listed in Appendix Table 9. Numbers on tree branches indicate the numbers of mutations associated with each branch. Numbers in brackets indicate clade 1 consensus mutations not detected, probably because of poor read coverage. Asterisks indicate samples that appear in both subclades 1a and 1b and are inferred to be a mixture of at >2 B.1.351 subtypes. Wastewater sequences ALB-Inf-04-21-21-A and COR-26th-04-04-21-A are not shown because several tracts of those sequences were too uncertain to enable accurate placement on the tree. OR, Oregon; BC, Benton County; CC, Clackamas County; LC, Linn County; MC, Multnomah County; WC, Washington County; DC, Washington, DC; SW, Switzerland.

*Sequences from individual clinical specimens are identified by their GISAID accession numbers (https://www.gisaid.org). Wastewater sequences are identified by their field collection identifier. Co., County; +, mutation detected; –, mutation not detected; ?, inadequate sequence data for a determination. †Cities where individual clinical specimens were collected are not provided to reduce identifiability of case-patients. ‡Number of mutations matched by the sequences from each sample. Mutations specific to B.1.351 are G174T, A2692T, G5230T, A21801C, 22283∆9, G22813T, C25904T, C26456T, and C28253T. Mutations specific to clade 1 are A1763G, C5100T, G13045A, C19524T, 28027∆129, and C29741T. §A single mutation defines each of clades 1a (A11875G) and 1b (C15928T). Absence of both mutations defines clade 1c in the case of individual specimens; in the case of wastewater samples, determining whether a mutation is truly absent from the RNA molecules present, or if the mutations have simply not been detected, is not possible. ¶This sample was retrospectively identified as B.1.351 late in April 2021 after routine sequencing of historical specimens. #This specimen falls into clade 2 (Figure 1).

Upon receipt of the samples, we subjected the filters to bead beating and extracted RNA by using the MagMAX Viral/Pathogen Nucleic Acid Isolation Kit (Thermo Fisher Scientific, https://www.thermofisher.com). We quantified 2 SARS-CoV-2 gene targets (nucleocapsid gene 1 and 2) and a human gene target (ribonuclease P, an internal control) through droplet digital reverse transcription PCR (ddRT-PCR) on a QX200 ddPCR system (Bio-Rad Labatories, ttps://www.bio-rad.com) using the 2019-nCoV CDC ddPCR Triplex Probe Assay (Bio-Rad) and the One-Step RT-ddPCR Advanced Kit for Probes (Bio-Rad), according to the manufacturer’s protocols.

### Amplicon-Based Sequencing

We performed amplicon-based sequencing to enable high coverage for the length of the genome, except 25 bp at each end. We synthesized cDNA by using the SuperScript IV First-Strand Synthesis System (Thermo Fisher) and sequenced it by using the Swift Amplicon SARS-CoV-2 Panel (Swift Biosciences, https://www.idtdna.com), together with Swift Amplicon Combinatorial Dual indexed adapters (Swift Biosciences), according to the manufacturer’s protocols. The Swift Amplicon SARS-CoV-2 Panel spans the SARS-CoV-2 genome with 341 amplicons with an average length of 150 bp. We produced sequences on a HiSeq 3000 or NextSeq 2000 sequencer (Illumina, https://www.illumina.com) to a depth sufficient for confident identification of variants, typically 10–30 million sequence reads per wastewater sample (Appendix).

### Bioinformatic Processing of Sequences

We demultiplexed the sequence reads with zero index mismatches by using bcl2fastq2 version 2.20 (Illumina) for samples sequenced on the HiSeq 3000 and BCL Convert version 1.2.1 (Illumina) for the NextSeq 2000, then trimmed them by using BBDuk (BBMap version 38.84 (US Department of Energy Joint Genome Institute, https://jgi.doe.gov). We aligned trimmed reads to the reference sequence (Wuhan-Hu-1, GenBank accession no. NC_045512.2) by using the BWA-MEM algorithm version 0.7.17-r1188 (https://github.com/lh3/bwa) and coordinate-sorted them with SAMtools version 1.10 (Genome Research Limited, https://www.sanger.ac.uk); we then removed primer sequences by using Primerclip version 0.3.8 (Swift BioSciences). We converted reads from sam files to bam files and coordinate-sorted and indexed them using SAMtools. We then used GATK version 4.2.0.0 (Broad Institute, https://www.broadinstitute.org) to identify mutations compared with the reference sequence (Appendix). We used Integrated Genomics Viewer version 2.8.7 (Broad Institute) to manually inspect sequence alignments and mutation calls (*21*,*22*).

### Multilocus Sequence Typing

Through the well-established process of MLST (*12*–*18*), we matched sets of mutations unique to known SARS-CoV-2 variants to mutations found in the wastewater sequences to infer the presence of variants in the community’s wastewater. Because of the heterogenous nature of wastewater and the potential presence of numerous SARS-CoV-2 variants in wastewater, we used a set of mutations identified as specific to B.1.351 in Oregon to screen for this variant (Table; Appendix Figure 1). To create this unique panel, we screened a set of 22 mutations associated with B.1.351 (H. Tegally et al., unpub. data, https://doi.org/10.1101/2020.12.21.20248640) (Appendix) against a database of mutations associated with individual clinical specimens sequenced in Oregon and deposited into GISAID (*20*) and from published reports of novel variants (*23*–*28*) (I. Ferreira, unpub. data, https://doi.org/10.1101/2021.05.08.443253; X. Deng et al., unpub. data, https://doi.org/10.1101/2021.03.07.21252647; M.K. Annavajhala et al., unpub. data, https://doi.org/10.1101/2021.02.23.21252259). Of the 22 mutations associated with the B.1.351 variant, 9 were found only in identified B.1.351 sequences from Oregon, 7 were common to >20 variants, and the remaining 6 were shared by 1–3 other variants (Appendix Tables 2–8, Figure 1).

Because wastewater SARS-CoV-2 RNA is derived from a mixture of variants, sequence reads spanning a B.1.351 mutation site would be expected to include reads derived from B.1.351 RNA molecules as well as reads derived from other variants. For a potential positive identification of a B.1.351-associated mutation in wastewater RNA sequences, a lower limit of 5% of sequence reads (with a minimum of 6 total reads) spanning the mutation site was required. In addition, >2 different sites carrying B.1.351 mutations must have been present. The rate of sequence errors produced by the sequencing procedure (a possible source of false-positive reads) was <0.2%.

Ultimately, we used 2 criteria to identify wastewater sample matches to B.1.351: the number of unique mutations present and the normalized proportion of all sequence reads. A minimum of 5 of 9 unique mutations was required for positive identification and we assigned a confidence score as follows: 8–9 matches indicated confident detection, 6–7 matches indicated probable detection, and 5 matches indicated tentative detection. In addition, the normalized proportion of all reads carrying any of the 9 mutations was required to be >10% of all reads spanning the 9 mutation sites (Appendix). We reconstructed the putative genome sequences of all variants inferred to be present in wastewater by mapping detected variant-specific mutations onto the reference sequence. We visualized and compared clinical specimen genomes from GISAID and putative viral isolate genomes by using Nextclade version 1.5.2 and used the output to manually construct the maximum-parsimony tree (*29*) (Figure 1). We submitted all wastewater sequences to the National Center for Biotechnology Information Sequence Read Archive (https://www.ncbi.nlm.nih.gov/sra) (*30*).

### Phylogenetic Analysis

We constructed phylogenetic trees by using a maximum-parsimony approach (*29*). Because of the simple structure of the Linn County B.1.351 population, we were able to manually construct a single unambiguous tree by using the mutations specific for each clade and subclade together with the mutations private to each clinical sequence (Appendix Table 9). Mutations private to B.1.351 sequences in wastewater could not be reliably identified because of the presence of other variants, so we did not include them in phylogenetic analysis.

The manual approach enabled us to include sequences that had mutation information missing because of poor sequencing quality. We first constructed the tree by using sequences missing no mutations. Then, we added sequences with missing data to the tree on the basis of available mutation data; this approach imputed the presence of the undetected mutations on the basis of the presence of the available mutations. We did not include in the trees sequences that could not be unambiguously placed on the tree due to missing data.

## Results

**Figure 2:**
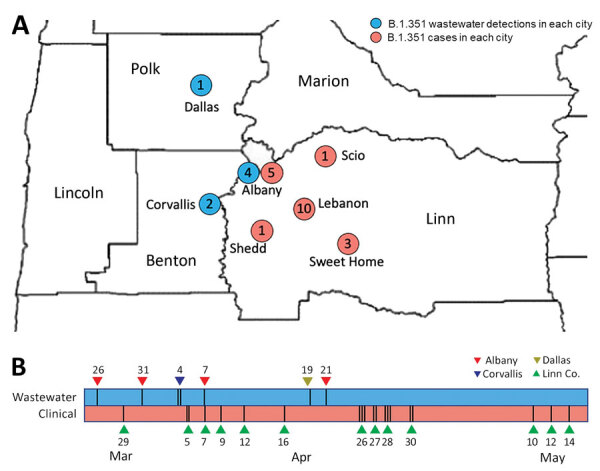
Location and timeline of emergence of SARS-CoV-2 variant B.1.351 in wastewater samples and clinical specimens in Linn County, Oregon, USA, and surrounding jurisdictions, March–May 2021.  A) Blue dots represent the sites and numbers of wastewater samples with detections of the B.1.351 variant in Linn County and surrounding jurisdictions. Red dots represent the location and number of individual cases of B.1.351 in Linn County. Initial wastewater samples with evidence of the B.1.351 variant of concern were collected from Albany, Oregon, during March 26–31, 2021, and the first case of B.1.351 infection in Linn County was reported on April 23, 2021 (specimen collection date of April 7, 2021); 18 additional cases were identified through May 15, 2021, including cases with earlier specimen collection dates. B) Timeline of wastewater samples and clinical specimens positive for B.1.351 in Linn County and surrounding jurisdictions. Vertical bars indicate the number of samples or specimens collected on each date. City locations are not given to limit identifiability of individual case-patients.

On March 26 and March 31, 2021, routine wastewater surveillance from the city of Albany, Oregon (Linn County), identified 2 samples that contained SARS-CoV-2 RNA exhibiting mutations specific to the B.1.351 lineage in Oregon (Table; Appendix Figure 1). The wastewater sample from March 26 (ALB-Inf-03–26–21-A) exhibited 7 of 9 specific mutations, whereas the wastewater sample from March 31 (ALB-Inf-03–31–21-A) exhibited all 9. At the time of these initial wastewater detections, no cases of B.1.351 in Linn County or adjacent counties had been identified, and only 25 specimens had been identified as B.1.351 through individual-level whole-genome sequencing statewide. On April 23, 2021, the first case of B.1.351 in Linn County was reported to the local public health authority (specimen collection date April 7). During the following month, 15 additional cases were reported. In Linn County, 20 total cases were identified through sequencing of individual clinical specimens collected through May 15 (Figure 2).

Additional community-level evidence in support of the initial detection of B.1.351 in the wastewater of Albany came from wastewater surveillance in 2 nearby cities as well as subsequent wastewater specimens from Albany. On April 4 and April 19, 2021, routine wastewater surveillance and sequencing of samples from the cities of Corvallis (Benton County; samples COR-25th-04-04-21-A and COR-26th-04-04-21-A) and Dallas (Polk County; sample DAL-Inf-04-19-21-A) identified probable (6/9) to confident (9/9) matches to the unique set of B.1.351 mutations referenced previously (Table; Appendix Figure 1), consistent with local circulation of the B.1.351 variant. Subsequent wastewater surveillance in Albany on April 7 (ALB-Inf-04-07-21-A) and April 21 (ALB-Inf-04-21-21-A) contained confident (8/9) and tentative (5/9) matches to the set of nine unique mutations. In sum, 7 wastewater samples matched >5 of the 9 mutations unique to the B.1.351 lineage (Table; Appendix Figure 1). In some cases, the lack of confident matches resulted from poor sequence coverage (<6 reads), whereas in other cases, no match was detected despite moderate sequence coverage (Appendix Figure 1).

Individual-level sequencing results were available for 19 of the 20 B.1.351 specimens identified in Linn County through May 15, 2021, in GISAID. Phylogenetic analysis of all 25 Oregon B.1.351 sequences available in GISAID through March 31, 2021, revealed 3 distinct B.1.351 clades in Oregon (Figure 1). Of the 19 Linn County specimens, 18 resided within a single clade (clade 1) defined by 6 unique mutations (A1763G, C5100T, G13045A, C19524T, 28027∆129, and C29741T), whereas 1 resided within a second clade defined by a distinct set of 6 mutations (clade 2). Two additional mutations divided clade 1 into 3 subclades: subclade 1a (14 sequences, defined by A11875G), subclade 1b (2 sequences, defined by C15928T), and subclade 1c (2 sequences, defined by neither mutation).

To assess the reliability with which B.1.351 was inferred to be present in the wastewater samples, and to genetically relate the wastewater samples to the individual clinical specimens, we searched the wastewater sequences from Albany and the nearby cities of Corvallis and Dallas for matches to the additional mutations identified in the individual specimens. For the 6 mutations defining clade 1, 6 of the 7 wastewater sequences matched >3 mutations and 3 matched all 6 mutation sites (Table; Appendix Figure 1). Three wastewater samples matched clade 1a (defined by mutation A11875G), and 4 samples matched clade 1b (defined by mutation C15928T). Two wastewater samples from Albany (ALB-Inf-03-30-21-A and ALB-Inf-04-07-21-A) matched both mutations, suggesting that those samples contained a mixture of SARS-CoV-2 RNA from both subclades. None of the wastewater samples exhibited mutations characteristic of clade 2, which included only 1 individual clinical specimen. The matches of the wastewater sequences to the additional mutations specific to clade 1 found in most Linn County cases substantially increase the confidence that B.1.351 RNA sequences were correctly identified in the wastewater samples.

We further assessed the sequences of each of the 7 wastewater samples for the presence of 6 additional mutations characteristic of B.1.351 but shared by other variants, including B.1.1.7, B.1.526, and P.1 (Appendix Table 8). To determine if interfering variants were present, we screened the sequences of each wastewater sample for mutations unique to those variants. We did not detect interfering variants in either of the 2 initial wastewater samples (ALB-Inf-03-26-21-A and ALB-03-30-21-A), and both samples exhibited all 6 of the additional shared mutations (Appendix Figure 1). These results provide further evidence for the true identification of B.1.351 in the initial 2 wastewater samples collected in Albany. The remaining 5 wastewater samples from Albany (ALB-Inf-04-07-21-A and ALB-Inf-04-21-21-A), Corvallis (COR-25th-04-04-21-A and COR-26th-04-04-21-A), and Dallas (DAL-Inf-04-19-21-A) demonstrated mutations consistent with B.1.1.7 and, in 1 case, P.1 (Appendix Tables 2–7, Figure 1).

## Discussion

In late March 2021, routine sequencing of SARS-CoV-2 wastewater surveillance detected the emergence of the B.1.351 (Beta) variant of concern in rural Linn County, Oregon, before its identification in individual cases. Currently, wastewater surveillance is used to track SARS-CoV-2 transmission trends in several jurisdictions and, in times of minimal transmission, may serve as an early warning system for disease resurgence (*31*). Wastewater surveillance offers local public health authorities and communities actionable data that is independent of symptomatic infection, healthcare access, and testing acceptance and may help in developing vaccination strategy (*32*). Leveraging this surveillance to support genomic surveillance for SARS-CoV-2 offers cost-effective community-level surveillance that may detect not only prevalent circulating variants but emerging variants of concern as well.

Accurate interpretation of wastewater sequencing results faces several challenges. These challenges include the heterogeneous nature of wastewater samples, the fragmentation of viral RNA in wastewater, the need to match wastewater sequences to panels of mutations characteristic of known variants, the variable levels of variant RNA in wastewater samples, the uneven sequence coverage of the viral genome in wastewater sequences, and the sharing of mutations (e.g., N501Y and E484K) across multiple variants. We used the well-established approach of MLST (*13*,*18*) in a novel application to infer the presence of RNA from SARS-CoV-2 variants in community wastewater samples from a statewide wastewater surveillance program.

MLST has been used to detect other pathogens in complex environmental samples, including wastewater. In addition, MLST has been used to analyze fragmented genetic molecules through the rigorous identification of matches to a curated set of mutations (i.e., a mutation panel) (*12*,*13*). Confidence in a detection is based on the proportion of matches to the mutation panel. Amplicon-based sequencing with the Swift Amplicon SARS-CoV-2 Panel is well-suited to MLST, providing excellent coverage of the entire SARS-CoV-2 genome, omitting only 25 bp at each end. With 341 overlapping amplicons of 150 bp on average, this method is robust to most mutations that could disrupt the binding of a primer (i.e., cause primer dropout) (N.L. Washington et al., unpub. data, https://doi.org/10.1101/2020.12.24.20248814).

To establish mutation panels suitable for screening for individual variants, we began with the canonical mutations defining each variant, derived either from the literature (*24–27,29,32*; I. Ferreira, unpub. data; X. Deng et al., unpub. data; M.K. Annavajhala et al., unpub. data) or from the Centers for Disease Control and Prevention (*33*). These sets of mutations were validated through the creation of an expanded panel of mutations identified in statewide individual sequencing data submitted to GISAID. Finally, all mutations shared with known variants were filtered out. This validation step would not be available to an emerging variant for which no local or regional individual-level sequencing data were available, highlighting the complementary properties of individual-level and community-level surveillance.

To address variable levels of variant RNA in the wastewater samples, we conducted in-depth sequencing, producing ≈10–30 million sequence reads per sample, to obtain sufficient sequence data to detect variants comprising as little as 10% of the RNA, even from samples with the lowest levels of viral RNA (log_10_ gene copies/L of 4.0). To address uneven sequence coverage of the viral genome in wastewater sequences, ranging from <10 to >1,000 reads per amplicon within a single sample, the sequence coverage was normalized to 100 reads per site for mutation sites with coverage of >100 reads. For sites with coverage of <100 reads per site, the actual read numbers were used to assign a proportionately smaller weight to those more poorly sequenced mutation sites.

In this study, we used a panel of 9 mutations identified as specific to B.1.351 to screen for the presence of this emerging variant of concern. We then used a subsequent panel of 8 additional mutations defining a single clade of B.1.351 sequences identified through statewide individual specimen sequencing to validate the initial set of matches, together with a case-by-case examination of a set of 6 mutations characteristic of B.1.351 but shared with other variants. This 2-step process of screening followed by validation, together with the large number of mutations within the screening and validation panels, rendered the detection of B.1.351 robust to small numbers of mismatches that occurred because of low sequence coverage, low levels of variant RNA, or primer dropout. The ability to compare independent but geographically or temporally related wastewater samples with closely related individual sequences substantially increased confidence in our detection of B.1.351 through wastewater surveillance (Figure 2).

All 7 wastewater sequences and 18 of 19 B.1.351 individual clinical specimen sequences clustered into a single clade (clade 1). The similarity of the sequences and their spatiotemporal proximity suggests a single common origin of the detected viruses. The SARS-CoV-2 sequences most closely related to the sequences in clade 1 were found in Switzerland (Figure 1), suggesting that the Oregon clade 1 cluster in Linn County may have originated from outside the United States. Even though B.1.351 was detected in the wastewater of the nearby cities of Corvallis (Benton County; OSU-25th-04-04-21-A) and Dallas (Polk County; DAL-Inf-04-19-21-A), no cases were identified in Benton or Polk Counties during this period. Thus, the detection of B.1.351 in these 2 counties may have resulted from the transient presence of cases from neighboring counties or may simply reflect insufficient individual-level genomic surveillance to detect B.1.351 in those areas.

Together, the complementary wastewater and clinical data we present clearly support community transmission of the B.1.351 variant in the Linn County region from late March through mid-May 2021. Wastewater sampling detected this emerging variant of concern 12 days before the specimen collection date of the first local case-patient. Wastewater surveillance therefore may be an efficient and reliable means of community-level monitoring for emerging SARS-CoV-2 variants and other human pathogens. Additional studies such as ours must be replicated across rural and urban settings to further understanding of the generalizability and limitations of wastewater surveillance. Scientific consensus regarding methods and minimum thresholds for variant detection in wastewater are urgently needed. 

Appendix Additional information about detection of SARS-CoV-2 B.1.351 (Beta) variant through wastewater surveillance before case detection in a community, Oregon.
