# Supplementary material for: Detection of SARS-CoV-2 B.1.351 (Beta) Variant through Wastewater Surveillance before Case Detection in a Community, Oregon, USA
Source: Emerg Infect Dis. 2022 Jun;28(6):1101–9. doi: 10.3201/eid2806.211821 (PMC9155900; doi:10.3201/eid2806.211821)
Supplement: Appendix — Additional information about detection of SARS-CoV-2 B.1.351 (Beta) variant through wastewater surveillance before case detection in a community, Oregon. [file 21-1821-Techapp-s1.pdf]

Article DOI: https://doi.org/10.3201/eid2806.211821

Detection of SARS-CoV-2 B.1.351 (Beta) Variant through Wastewater Surveillance before Case Detection in a Community, Oregon, USA

Appendix

Methods

Sequencing Depth and Quality Assurance

The percentage of sequence reads that aligned to the SARS-CoV-2 reference genome were based principally on the SARS-CoV-2 RNA concentration in wastewater samples. RNA concentrations of $\log_{10}$ gene copies/liter of 4.0 to 5.0 typically yielded 0.1% to 5.0% alignable reads (Appendix Table 1) while RNA concentrations over 7.0 have yielded over 90% alignable reads. In contrast, RNA concentrations below 4.0 produced poor results with the methodology described here, with many Swift amplicons not represented at all among the sequences (data not shown). Best results with MLST, with most mutation sites receiving over 10 sequence reads, were generally obtained when the number of aligned reads was 20,000 or more. To achieve this depth for most samples, a total of 10 to 30 million raw reads per sample were typically required and this was achieved by adjusting the number of sequencing lanes on the Illumina HiSeq3000 to the number of samples in a sequencing batch. Generally, for up to 40 samples, one lane was used; for 40-70 samples, two lanes were used (Appendix Table 1), and for 70-96 samples, three lanes were used.

Negative controls lacking RNA and positive controls containing synthetic SARS-CoV-2 RNA (Twist Biosciences, San Francisco, CA; Controls 12 and 13, parts #103515 and103533) were sequenced with each batch of wastewater samples. The negative controls were used to check for cross-contamination. The positive controls containing synthetic RNA were utilized to assess sequence quality and sensitivity. To minimize the risk of cross-contamination, RNA from nasal swabs, which typically contained high concentrations of SARS-CoV-2 RNA, were

sequenced on different days and analyzed in different Illumina HiSeq3000 lanes than wastewater samples. Sets of samples with failed negative or positive controls were re-sequenced. All data reported here are from samples that passed these quality control tests.

Identification of Mutations Using GATK Software

The GATK software package (version 4.2.0.0, Broad Institute, Cambridge, MA) was used to identify mutations in wastewater sequence reads compared to the reference sequence using the following procedure: variants were called on a per sample basis using GATK's HaplotypeCaller sub-package with the following settings: -stand-call-conf 20, --dont-use-soft-clipped-bases, -mbq 20, --max-reads-per-alignment-start 0, --linked-de-bruijn-graph, --recover-all-dangling-branches, --sample-ploidy 4. gVCF files were merged using GATK's CombineGVCFs sub-package and jointly called with GATK's GenotypeGVCFs sub-package while forcing the output of the standard loci. The final VCF file was converted to a tabular format with GATK's VariantsToTable sub-package.

Establishment of the B.1.351 Mutation Panel for MLST

The B.1.351 mutation panel was initially based on the 18 substitution mutations appearing in greater than 90% of the 190 B.1.351 sequences originally described (23), namely G174T, C241T, C1059T, A2692T, C3037T, G5230T, A10323G, C14408T, A21801C, G22813T, G23012A, A23063T, A23403G, C23664T, C25904T, C26456T, G25563T, C28253T, and C28887T. Subsequent examination of 46 U.S. B.1.351 sequences collected through December 31, 2020 and deposited in GISAID, suggested that four additional mutations had become fixed in the U.S. B.1.351 population, namely A2692T, 11288Δ9, A22206G, and 22283Δ9. These mutations were, therefore, added to the screening panel.

The following six mutations were then removed from the panel due to their co-occurrence in numerous other B.1 lineages: C241T, C1059T, C3037T, C14408T, A23403G, G25563T, and C28887T. Finally, another set of six mutations, A10323G, 11288Δ9, A22206G, G23012A, A23063T, and C23664T, were removed from the panel because they also occurred in other published lineages (B.1.1.7, B.1.1.316, B.1.526, and P.1) or in lineages encountered in sequences from Oregon individuals (B.1.404, B.1.582) (Appendix Table 8). We refer to such lineages as "interfering variants".

In order to detect the possible occurrence of emerging (i.e., previously undefined) interfering variants with mutations overlapping with the B.1.351 panel, we routinely screened for mutations from the B.1.351 panel that were present in the absence of matches to any other mutations in the B.1.351 panel. Sequences exhibiting such mutations were then clustered to determine if there were additional mutations co-occurring with the mutation in question. If clusters of co-occurring mutations were observed, we inferred the possible presence of a novel interfering variant and removed the mutation from the panel. Although this procedure did not detect novel variants that interfered with the detection of B.1.351, the procedure regularly identified novel putative variants interfering with detection of other variants in our broader screening panel.

Estimating the Fraction of RNA Molecules Attributable to B.1.351 in Samples

One criterion used to assess the reliable detection of B.1.351 in a wastewater sample was an estimated percentage of RNA molecules in the sample attributable to B.1.351 greater than 10%, based on information from all the mutations in the screening panel. This quantitation was challenging because the number of sequence reads spanning the nine unique mutation sites could vary over a 250-fold range, due to differing amplification and sequencing efficiencies of the 341 Swift amplicons (Appendix Tables 2–7). We first calculated the fraction of all reads spanning a mutation site that exhibited the mutation and averaged the ratios over all nine mutation sites, however, this approach proved unsatisfactory as mutation sites with low read coverage exhibited highly variable ratios which biased the estimate. We then summed the number of all reads exhibiting any of the nine mutations and divided by the number of reads spanning all the mutation sites, however, this approach also proved unsatisfactory as the estimate was biased by the most deeply sequenced mutation sites. A hybrid method was, therefore, adopted. For mutation sites with more than 100 sequence reads, the numbers of reads exhibiting the mutation were normalized to a total of 100 reads (Appendix Tables 2–7). For mutation sites with fewer than 100 sequence reads, the actual numbers of reads were retained. The normalized numbers of all reads exhibiting any of the nine mutations were then summed and divided by the sum of the normalized numbers of reads spanning all the mutation sites (Appendix Tables 2–7).

When all sites were spanned by more than 100 reads, the ratio produced by the hybrid method was identical to that produced by the first approach of averaging the ratios at the nine sites. Through the hybrid method, when some sites had less than 100 reads, their contribution to

the final estimate was reduced in proportion to their lower read coverage. The hybrid method of estimation reduced bias from both under-sequenced and over-sequenced sites. All samples identified in this paper as including B.1.351 RNA sequences exhibited estimates over the minimum threshold of 0.1 (Appendix Tables 2–7).

Appendix Table 1. Sequencing statistics for wastewater samples

Sample	SARS-CoV-2 RNA*	Samples/Lanes†	Passing reads‡	Aligned reads§	% Aligned§
ALB-Inf-3-26-2021-A	4.50	69/2 HiSeq	14,545,578	19,576	0.13%
ALB-Inf-3-31-2021-A	4.54	69/2 HiSeq	12,916,288	23,610	0.18%
ALB-Inf-4-7-2021-A	4.53	58/2 HiSeq	17,755,758	82,631	0.46%
ALB-Inf-4-14-2021-A¶	4.77	49/2 HiSeq	26,941,183	1,319,703	4.90%
ALB-Inf-4-21-2021-A	4.64	57/2 HiSeq	12,504,849	47,703	0.38%
DAL-Inf-4-19-2021-A	5.04	57/2 HiSeq	18,047,175	558,391	3.09%
COR-25TH-4-4-21-A	4.96	69/2 HiSeq	21,511,528	281,282	1.31%
COR-26TH-4-4-21-A	4.17	69/2 HiSeq	18,426,836	49,452	0.27%
COR-27TH-4-4-21-A¶	4.57	69/2 HiSeq	15,713,982	45,010	0.29%

* SARS-CoV-2 RNA concentrations in the original wastewater sample, as estimated by digital droplet PCR. Units are log_{10} gene copies/liter.
† Each sample was included in a batch of the indicated number of samples and sequence was generated from the batch using the indicated numbers of lanes of an Illumina HiSeq3000 instrument.
‡ Numbers of reads passing Illumina QC for each sample.
§ Number and percentage of passing reads aligning to the reference sequence for each sample.
¶ These samples were ultimately classified as no detection of B.1.351.

Appendix Table 2. Summary of sequence read analysis of mutations specific to B.1.351 in wastewater samples in Linn County, Oregon and surrounding jurisdictions, March-April 2021

Sample identifier*	Source	Collection Date	Final call†	Read fraction‡	Mutation count§
ALB-Inf-03-26-21-A	Albany wastewater plant	March 26, 2021	Probable	0.423	7/9
ALB-Inf-03-31-21-A	Albany wastewater plant	March 31, 2021	Confident	0.454	9/9
ALB-Inf-04-07-21-A	Albany wastewater plant	April 7, 2021	Confident	0.245	8/9
ALB-Inf-04-14-21-A	Albany wastewater plant	April 14, 2021	Not detected	0	0/9
ALB-Inf-04-21-21-A	Albany wastewater plant	April 21, 2021	Tentative	0.142	5/9
COR-25TH-04-04-21-A	Corvallis 25th St. sewer	April 4, 2021	Confident	0.901	9/9
COR-26TH-04-04-21-A	Corvallis 26th St. sewer	April 4, 2021	Probable	0.566	6/9
COR-27TH-04-04-21-A	Corvallis 27th St. sewer	April 4, 2021	Trace	0.146	3/9
DAL-Inf-04-19-21-A	Dallas wastewater plant	April 19, 2021	Confident	0.552	9/9

* Wastewater samples analyzed in this study including two additional samples, collected from the Albany wastewater plant on April 14, 2021, and from Corvallis 27th St. sewer on April 4, 2021. B.1.351 was not reliably detected in those additional samples, however, they are included here to illustrate the variant calling procedure.
† The final call as to whether B.1.351 was present was based on the proportion of mutant reads and the number of mutations detected that were specific to B.1.351 (out of 9). If the overall proportion of mutant reads was less than 0.1, or the number of individual mutations detected was less than 3/9, then the variant was called as "Not detected". If the overall proportion of mutant reads was ≥ 0.1, the variant was called by the number of individual mutations present: 3-4 as “Trace”, 5 as “Tentative”, 6-7 as “Probable” and 8-9 as “Confident”.
‡ The overall proportion of mutant reads supporting the presence of the variant was calculated as the sum of the normalized read numbers for the 9 B.1.351-specific mutation sites divided by the sum of all the normalized read numbers for the 9 sites.
§ The count was the number of B.1.351-specific mutations meeting detection criteria.

Appendix Table 3. Detailed sequence read analysis of mutations specific to B.1.351 in wastewater samples in Linn County, Oregon and surrounding jurisdictions, March-April 2021

		Mutations‡								
Wastewater samples*	Analysis†	G174T	A2692T	G5230T	A21801C	22283Δ9	G22813T	C25904T	C26456T	C28253T
ALB-Inf-03-26-21-A	All_reads-actual	784	37	86	27	66	1	8	20	27
	Unsupport_reads-actual	516	4	57	9	42	1	8	4	24
	Support_reads-actual	268	33	29	18	24	0	0	16	3
	All_reads-normalized	100	37	86	27	66	1	8	20	27
	Unsupport_reads-normalized	65.8	4.0	57	9	42	1	8	4	24
	Support_reads-normalized	34.2	33.0	29	18	24	0	0	16	3
	Proportion	0.342	0.892	0.337	0.667	0.364	0	0	0.800	0.111
	Mutation call	Present	Present	Present	Present	Present	Insuff	Absent	Present	Present
ALB-Inf-03-31-21-A	All_reads-actual	617	38	189	115	62	16	27	44	116
	Unsupport_reads-actual	303	12	138	34	42	6	5	14	104
	Support_reads-actual	314	26	51	81	20	10	22	30	12
	All_reads-normalized	100	38	100	100	62	16	27	44	100
	Unsupport_reads-normalized	49.1	12.0	73.0	29.6	42	6	5	14	89.7
	Support_reads-normalized	50.9	26.0	27.0	70.4	20	10	22	30	10.3
	Proportion	0.509	0.684	0.270	0.704	0.323	0.625	0.815	0.682	0.103
	Mutation call	Present	Present	Present	Present	Present	Present	Present	Present	Present
ALB-Inf-04-07-21-A	All_reads-actual	4472	247	562	263	162	45	40	109	294
	Unsupport_reads-actual	3370	148	431	196	113	27	38	106	219
	Support_reads-actual	1102	99	131	67	49	18	2	3	75
	All_reads-normalized	100	100	100	100	100	45	40	100	100
	Unsupport_reads-normalized	75.4	59.9	76.7	74.5	69.8	27	38	97.2	74.5
	Support_reads-normalized	24.6	40.1	23.3	25.5	30.2	18	2	2.8	25.5
	Proportion	0.246	0.401	0.233	0.255	0.302	0.400	0.050	0.028	0.255
	Mutation call	Present	Present	Present	Present	Present	Present	Present	Absent	Present
ALB-Inf-04-14-21-A	All_reads-actual	23135	2732	1788	2682	2807	945	1699	241	649
	Unsupport_reads-actual	23135	2732	1788	2682	2807	945	1699	241	649
	Support_reads-actual	0	0	0	0	0	0	0	0	0
	All_reads-normalized	100	100	100	100	100	100	100	100	100
	Unsupport_reads-normalized	100	100	100	100	100	100	100	100	100
	Support_reads-normalized	0	0	0	0	0	0	0	0	0
	Proportion	0	0	0	0	0	0	0	0	0
	Mutation call	Absent	Absent	Absent	Absent	Absent	Absent	Absent	Absent	Absent
ALB-Inf-04-21-21-A	All_reads-actual	1208	197	51	120	241	2	11	42	90
	Unsupport_reads-actual	1123	186	51	101	217	2	11	42	44
	Support_reads-actual	85	11	0	19	24	0	0	0	46
	All_reads-normalized	100	100	51	100	100	2	11	42	90
	Unsupport_reads-normalized	93.0	94.4	51	84.2	90.0	2	11	42	44
	Support_reads-normalized	7.0	5.6	0	15.8	10.0	0	0	0	46
	Proportion	0.070	0.056	0	0.158	0.100	0	0	0	0.511
	Mutation call	Present	Present	Absent	Present	Present	Insuff	Absent	Absent	Present
COR-25TH-04-04-21-A	All_reads-actual	18138	829	1924	1046	737	146	204	339	493
	Unsupport_reads-actual	1585	70	151	10	145	12	38	16	59
	Support_reads-actual	16553	759	1773	1036	592	134	166	323	434
	All_reads-normalized	100	100	100	100	100	100	100	100	100
	Unsupport_reads-normalized	8.7	8.4	7.8	1.0	19.7	8.2	18.6	4.7	12.0
	Support_reads-normalized	91.3	91.6	92.2	99.0	80.3	91.8	81.4	95.3	88.0
	Proportion	0.913	0.916	0.922	0.990	0.803	0.918	0.814	0.953	0.880

		Mutations‡								
Wastewater samples*	Analysis†	G174T	A2692T	G5230T	A21801C	22283∆9	G22813T	C25904T	C26456T	C28253T
	Mutation call	Present	Present	Present	Present	Present	Present	Present	Present	Present
COR-26TH-04-04-21-A	All_reads-actual	707	40	288	186	56	0	57	20	191
	Unsupport_reads-actual	63	9	29	186	56	0	36	16	24
	Support_reads-actual	644	31	259	0	0	0	21	4	167
	All_reads-normalized	100	40	100	100	56	0	57	20	100
	Unsupport_reads-normalized	8.9	9.0	10.1	100	56	0	36	16	12.6
	Support_reads-normalized	91.1	31.0	89.9	0	0	0	21	4	87.4
	Proportion	0.911	0.775	0.899	0	0	0	0.368	0.200	0.874
	Mutation call	Present	Present	Present	Absent	Absent	Insuff	Present	Present	Present
COR-27TH-04-04-21-A	All_reads-actual	133	12	52	66	57	4	6	0	58
	Unsupport_reads-actual	133	7	52	49	30	1	6	0	58
	Support_reads-actual	0	5	0	17	27	3	0	0	0
	All_reads-normalized	100	12	52	66	57	4	6	0	58
	Unsupport_reads-normalized	100	7	52	49	30	1	6	0	58
	Support_reads-normalized	0	5	0	17	27	3	0	0	0
	Proportion	0	0.4166667	0	0.258	0.474	0.750	0	0	0
	Mutation call	Absent	Present	Absent	Present	Present	Insuff	Absent	Insuff	Absent
DAL-Inf-04-19-21-A	All_reads-actual	22203	1650	2192	2738	1761	402	1318	924	2370
	Unsupport_reads-actual	11120	768	924	1729	461	174	742	277	1084
	Support_reads-actual	11083	882	1268	1009	1300	228	576	647	1286
	All_reads-normalized	100	100	100	100	100	100	100	100	100
	Unsupport_reads-normalized	50.1	46.5	42.2	63.1	26.2	43.3	56.3	30.0	45.7
	Support_reads-normalized	49.9	53.5	57.8	36.9	73.8	56.7	43.7	70.0	54.3
	Proportion	0.499	0.535	0.578	0.369	0.738	0.567	0.437	0.700	0.543
	Mutation call	Present	Present	Present	Present	Present	Present	Present	Present	Present

* See Appendix Table 2

† Actual numbers of sequence reads spanning the mutation site (All_reads), the numbers of reads not supporting the presence of the mutation (Unsupport, the reference sequence), and the number of reads supporting the presence of the mutation (Support, mutant sequence) are shown. In addition, the normalized numbers of reads are shown. For normalization, read numbers greater than 100 were reduced proportionately to a total of 100 reads. For read numbers of 100 or less, the actual read number was retained. "Proportion" is the proportion of all reads that carry the mutation. Mutations were called present if the proportion was 0.05 or greater and the number of reads carrying the mutation was 2 or more. Otherwise, the mutation was called Absent. If the total number of reads was 5 or less, the call was "insufficient data" (Insuff).

‡ Mutations found only in B.1.351. See Figure 1 and Appendix Figure 1.

Appendix Table 4. Detailed sequence read analysis of mutations specific to B.1.351 clade 1 in wastewater samples in Linn County, Oregon and surrounding jurisdictions, March-April 2021

		Mutations‡							
Wastewater samples*	Analysis†	A1763G	C5100T	G13045A	C19524T	28026∆129	C29741T	A11875G§	C15928T§
ALB-Inf-03-26-21-A	All_reads-actual	2	46	30	46	44	10	48	134
	Unsupport_reads-actual	2	30	27	43	44	10	48	86
	Support_reads-actual	0	16	3	3	0	0	0	48
	All_reads-normalized	2	46	30	46	44	10	48	100
	Unsupport_reads-normalized	2	30	27	43	44	10	48	64.2
	Support_reads-normalized	0	16	3	3	0	0	0	35.8
	Proportion	0	0.348	0.100	0.065	0	0	0	0.358
	Mutation call	Insuff	Present	Present	Present	Absent	Absent	Absent	Present
ALB-Inf-03-31-21-A	All_reads-actual	2	13	15	48	48	77	41	216
	Unsupport_reads-actual	2	8	4	15	48	15	14	187
	Support_reads-actual	0	5	11	33	0	62	27	29
	All_reads-normalized	2	13	15	48	48	77	41	100
	Unsupport_reads-normalized	2	8	4	15	48	15	14	86.6
	Support_reads-normalized	0	5	11	33	0	62	27	13.4
	Proportion	0	0.385	0.733	0.688	0	0.805	0.659	0.134
	Mutation call	Insuff	Present	Present	Present	Absent	Present	Present	Present
ALB-Inf-04-07-21-A	All_reads-actual	23	72	169	127	463	161	239	511
	Unsupport_reads-actual	17	67	118	111	463	147	211	450
	Support_reads-actual	6	5	51	16	0	14	28	61
	All_reads-normalized	23	72	100	100	100	100	100	100
	Unsupport_reads-normalized	17	67	69.8	87.4	100	91.3	88.3	88.1
	Support_reads-normalized	6	5	30.2	12.6	0	8.7	11.7	11.9
	Proportion	0.261	0.069	0.302	0.126	0	0.087	0.117	0.119
	Mutation call	Present	Present	Present	Present	Absent	Present	Present	Present
ALB-Inf-04-14-21-A	All_reads-actual	364	1788	1423	1459	12667	769	3720	2285
	Unsupport_reads-actual	364	1788	1423	1459	12667	769	3720	2285
	Support_reads-actual	0	0	0	0	0	0	0	0
	All_reads-normalized	100	100	100	100	100	100	100	100
	Unsupport_reads-normalized	100	100	100	100	100	100	100	100
	Support_reads-normalized	0	0	0	0	0	0	0	0
	Proportion	0	0	0	0	0	0	0	0
	Mutation call	Absent	Absent	Absent	Absent	Absent	Absent	Absent	Absent
ALB-Inf-04-21-21-A	All_reads-actual	4	61	107	65	444	81	117	243
	Unsupport_reads-actual	2	48	95	64	444	0	115	243
	Support_reads-actual	2	13	12	1	0	0	2	0
	All_reads-normalized	4	61	100	65	100	81	98.3	100
	Unsupport_reads-normalized	2	48	88.8	64	100	0	1.7	100
	Support_reads-normalized	2	13	11.2	1	0	0	0	0
	Proportion	0.500	0.213	0.112	0.015	0	0	0.017	0
	Mutation call	Insuff	Present	Present	Absent	Absent	Absent	Absent	Absent
COR-25TH-04-04-21-A	All_reads-actual	59	333	753	355	45	413	42	281
	Unsupport_reads-actual	0	23	28	0	45	31	14	281
	Support_reads-actual	59	310	725	355	0	382	28	0
	All_reads-normalized	59	100	100	100	45	100	42	100
	Unsupport_reads-normalized	0	6.9	3.7	0	45	7.5	14	100
	Support_reads-normalized	59	93.1	96.3	100	0	92.5	28	0
	Proportion	1.000	0.931	0.963	1.000	0	0.925	0.667	0

Wastewater samples*	Analysis†	Mutations‡							
		A1763G	C5100T	G13045A	C19524T	28026∆129	C29741T	A11875G§	C15928T§
	Mutation call	Present	Present	Present	Present	Absent	Present	Present	Absent
COR-26TH-04-04-21-A	All_reads-actual	22	52	44	154	228	1	77	46
	Unsupport_reads-actual	4	9	29	135	228	1	77	46
	Support_reads-actual	18	43	15	19	0	0	0	0
	All_reads-normalized	22	52	44	100	100	1	77	46
	Unsupport_reads-normalized	4	9	29	87.7	100	1	77	46
	Support_reads-normalized	18	43	15	12.3	0	0	0	0
	Proportion	0.818	0.827	0.341	0.123	0	0	0	0
	Mutation call	Present	Present	Present	Present	Absent	Insuff	Absent	Absent
COR-27TH-04-04-21-A	All_reads-actual	1	29	31	49	85	10	40	158
	Unsupport_reads-actual	1	29	20	19	85	10	40	158
	Support_reads-actual	0	0	11	30	0	0	0	0
	All_reads-normalized	1	29	31	49	85	10	40	100
	Unsupport_reads-normalized	1	29	20	19	85	10	40	100
	Support_reads-normalized	0	0	11	30	0	0	0	0
	Proportion	0	0	0.355	0.612	0	0	0	0
	Mutation call	Insuff	Absent	Present	Present	Absent	Absent	Absent	Absent
DAL-Inf-04-19-21-A	All_reads-actual	194	1051	1010	1555	3487	1632	1428	4004
	Unsupport_reads-actual	69	289	395	501	3487	347	1428	797
	Support_reads-actual	125	762	615	1054	0	1285	0	3207
	All_reads-normalized	100	100	100	100	100	100	100	100
	Unsupport_reads-normalized	35.6	27.5	39.1	32.2	100	21.3	100	19.9
	Support_reads-normalized	64.4	72.5	60.9	67.8	0	78.7	0	80.1
	Proportion	0.644	0.725	0.609	0.678	0	0.787	0	0.801
	Mutation call	Present	Present	Present	Present	Absent	Present	Absent	Present

* See Appendix Table 2

† Actual numbers of sequence reads spanning the mutation site (All_reads), the numbers of reads not supporting the presence of the mutation (Unsupport, the reference sequence), and the number of reads supporting the presence of the mutation (Support, mutant sequence) are shown. In addition, the normalized numbers of reads are shown. For normalization, read numbers greater than 100 were reduced proportionately to a total of 100 reads. For read numbers of 100 or less, the actual read number was retained. "Proportion" is the proportion of all reads that carry the mutation. Mutations were called present if the proportion was 0.05 or greater and the number of reads carrying the mutation was 2 or more. Otherwise, the mutation was called Absent. If the total number of reads was 5 or less, the call was "insufficient data" (Insuff).

‡ Mutations found only in clade 1 of B.1.351 as defined in this paper. See Figure 1 and Appendix Figure 1.

§ The mutations A11875G C15928T define sub-clades 1a and 1b respectively, within clade 1. Clade 1 sequences lacking both mutations are defined as clade 1c. See Figure 1.

Appendix Table 5. Detailed sequence read analysis of B.1.351 mutations shared with 1-3 other variants in wastewater samples in Linn County, Oregon and surrounding jurisdictions, March-April 2021

Wastewater samples*	Analysis†	Mutations‡					
		A10323G	11288Δ9	A22206G	G23012A	A23063T	C23664T
ALB-Inf-03-26-21-A	All_reads-actual	38	43	25	7	7	76
	Unsupport_reads-actual	25	22	9	5	5	29
	Support_reads-actual	13	21	16	2	2	47
	All_reads-normalized	38	43	25	7	7	76
	Unsupport_reads-normalized	25	22	9	5	5	29
	Support_reads-normalized	13	21	16	2	2	47
	Proportion	0.342	0.488	0.640	0.286	0.286	0.618
	Mutation call	Present	Present	Present	Present	Present	Present
ALB-Inf-03-31-21-A	All_reads-actual	33	71	38	6	6	136
	Unsupport_reads-actual	10	13	26	1	1	106
	Support_reads-actual	23	58	12	5	5	30
	All_reads-normalized	33	71	38	6	6	100
	Unsupport_reads-normalized	10	13	26	1	1	77.9
	Support_reads-normalized	23	58	12	5	5	22.1
	Proportion	0.697	0.817	0.316	0.833	0.833	0.221
	Mutation call	Present	Present	Present	Present	Present	Present
ALB-Inf-04-07-21-A	All_reads-actual	98	176	75	76	73	402
	Unsupport_reads-actual	79	108	42	19	15	267
	Support_reads-actual	19	68	33	57	58	135
	All_reads-normalized	98	100	75	76	73	100
	Unsupport_reads-normalized	79	61.4	42	19	15	66.4
	Support_reads-normalized	19	38.6	33	57	58	33.6
	Proportion	0.194	0.386	0.440	0.750	0.795	0.336
	Mutation call	Present	Present	Present	Present	Present	Present
ALB-Inf-04-14-21-A	All_reads-actual	3599	4035	401	945	977	10041
	Unsupport_reads-actual	1440	0	401	945	1	10041
	Support_reads-actual	2159	4035	0	0	976	0
	All_reads-normalized	100	100	100	100	100	100
	Unsupport_reads-normalized	40	0	100	100	0	100
	Support_reads-normalized	60.0	100	0	0	99.9	0
	Proportion	0.600	1.000	0	0	0.999	0
	Mutation call	Present	Present	Absent	Absent	Present	Absent
ALB-Inf-04-21-21-A	All_reads-actual	65	74	60	1	1	499
	Unsupport_reads-actual	53	20	44	1	1	499
	Support_reads-actual	12	54	16	0	0	0
	All_reads-normalized	65	74	60	1	1	100
	Unsupport_reads-normalized	53	20	44	1	1	100
	Support_reads-normalized	12	54	16	0	0	0
	Proportion	0.185	0.730	0.267	0	0	0
	Mutation call	Present	Present	Present	Insuff	Insuff	Absent
COR-25TH-04-04-21-A	All_reads-actual	440	966	293	219	218	1125
	Unsupport_reads-actual	6	3	0	9	0	78
	Support_reads-actual	434	963	293	210	218	1047
	All_reads-normalized	100	100	100	100	100	100
	Unsupport_reads-normalized	1.4	0.3	0	4.1	0	6.9
	Support_reads-normalized	98.6	99.7	100	95.9	100	93.1
	Proportion	0.986	0.997	1.000	0.959	1.000	0.931

Wastewater samples*	Analysis†	Mutations‡					
		A10323G	11288∆9	A22206G	G23012A	A23063T	C23664T
	Mutation call	Present	Present	Present	Present	Present	Present
COR-26TH-04-04-21-A	All_reads-actual	108	59	56	1	1	43
	Unsupport_reads-actual	55	28	56	1	1	43
	Support_reads-actual	53	31	0	0	0	0
	All_reads-normalized	100	59	56	1	1	43
	Unsupport_reads-normalized	50.9	28	56	1	1	43
	Support_reads-normalized	49.1	31	0	0	0	0
	Proportion	0.491	0.525	0	0	0	0
	Mutation call	Present	Present	Absent	Insuff	Insuff	Absent
COR-27TH-04-04-21-A	All_reads-actual	9	38	18	21	21	78
	Unsupport_reads-actual	6	24	8	21	3	31
	Support_reads-actual	3	14	10	0	18	47
	All_reads-normalized	9	38	18	21	21	78
	Unsupport_reads-normalized	6	24	8	21	3	31
	Support_reads-normalized	3	14	10	0	18	47
	Proportion	0.333	0.368	0.556	0	0.857	0.603
	Mutation call	Present	Present	Present	Absent	Present	Present
DAL-Inf-04-19-21-A	All_reads-actual	1095	1485	649	1215	1183	1895
	Unsupport_reads-actual	206	145	140	124	109	1122
	Support_reads-actual	889	1340	509	1091	1074	773
	All_reads-normalized	100	100	100	100	100	100
	Unsupport_reads-normalized	18.8	9.8	21.6	10.2	9.2	59.2
	Support_reads-normalized	81.2	90.2	78.4	89.8	90.8	40.8
	Proportion	0.812	0.902	0.784	0.898	0.908	0.408
	Mutation call	Present	Present	Present	Present	Present	Present

* See Appendix Table 2

† Actual numbers of sequence reads spanning the mutation site (All_reads), the numbers of reads not supporting the presence of the mutation (Unsupport, the reference sequence), and the number of reads supporting the presence of the mutation (Support, mutant sequence) are shown. In addition, the normalized numbers of reads are shown. For normalization, read numbers greater than 100 were reduced proportionately to a total of 100 reads. For read numbers of 100 or less, the actual read number was retained. "Proportion" is the proportion of all reads that carry the mutation. Mutations were called present if the proportion was 0.05 or greater and the number of reads carrying the mutation was 2 or more. Otherwise, the mutation was called Absent. If the total number of reads was 5 or less, the call was "insufficient data" (Insuff).

‡ Mutations found in B.1.351 sequences but also found in 1 to 3 other variants. See Appendix Table 8 for a list of variants that share these mutations.

Appendix Table 6. Detailed sequence read analysis of B.1.351 mutations shared with >20 other variants in wastewater samples in Linn County, Oregon and surrounding jurisdictions, March-April 2021

		Mutations‡						
Wastewater samples*	Analysis†	C241T	C1059T	C3037T	C14408T	A23403G	G25563T	C28887T
ALB-Inf-03-26-21-A	All_reads-actual	800	45	180	29	25	64	12
	Unsupport_reads-actual	4	0	0	0	0	20	5
	Support_reads-actual	796	45	180	29	25	44	7
	All_reads-normalized	100	45	100	29	25	64	12
	Unsupport_reads-normalized	0.5	0	0	0	0	20	5
	Support_reads-normalized	99.5	45	100	29	25	44	7
	Proportion	0.995	1.000	1.000	1.000	1.000	0.688	0.583
	Mutation call	Present	Present	Present	Present	Present	Present	Present
ALB-Inf-03-31-21-A	All_reads-actual	567	87	239	32	13	274	36
	Unsupport_reads-actual	0	0	0	0	0	0	0
	Support_reads-actual	567	87	239	32	13	274	36
	All_reads-normalized	100	87	100	32	13	100	36
	Unsupport_reads-normalized	0	0	0	0	0	0	0
	Support_reads-normalized	100	87	100	32	13	100	36
	Proportion	1.000	1.000	1.000	1.000	1.000	1.000	1.000
	Mutation call	Present	Present	Present	Present	Present	Present	Present
ALB-Inf-04-07-21-A	All_reads-actual	3662	379	649	139	78	439	283
	Unsupport_reads-actual	0	18	0	0	0	45	218
	Support_reads-actual	3662	361	649	139	78	394	65
	All_reads-normalized	100	100	100	100	78	100	100
	Unsupport_reads-normalized	0	4.7	0	0	0	10.3	77.0
	Support_reads-normalized	100	95.3	100	100	78	89.7	23.0
	Proportion	1.000	0.953	1.000	1.000	1.000	0.897	0.230
	Mutation call	Present	Present	Present	Present	Present	Present	Present
ALB-Inf-04-14-21-A	All_reads-actual	31849	2956	13349	1841	2525	7242	1990
	Unsupport_reads-actual	117	2688	1	0	3	7242	1990
	Support_reads-actual	31732	268	13348	1841	2522	0	0
	All_reads-normalized	100	100	100	100	100	100	100
	Unsupport_reads-normalized	0	91	0	0	0	100	100
	Support_reads-normalized	99.6	9.1	100.0	100	99.9	0	0
	Proportion	0.996	0.091	1.000	1.000	0.999	0	0
	Mutation call	Present	Present	Present	Present	Present	Absent	Absent
ALB-Inf-04-21-21-A	All_reads-actual	1126	85	362	83	35	626	78
	Unsupport_reads-actual	1	19	0	0	0	549	59
	Support_reads-actual	1125	66	362	83	35	77	19
	All_reads-normalized	100	85	100	83	35	100	78
	Unsupport_reads-normalized	0.1	19	0	0	0	87.7	59
	Support_reads-normalized	99.9	66	100	83	35	12.3	19
	Proportion	0.999	0.776	1.000	1.000	1.000	0.123	0.244
	Mutation call	Present	Present	Present	Present	Present	Present	Present
COR-25TH-04-04-21-A	All_reads-actual	11695	1247	3461	566	347	1321	292
	Unsupport_reads-actual	8	37	3	0	0	31	10
	Support_reads-actual	11687	1210	3458	566	347	1290	282
	All_reads-normalized	100	100	100	100	100	100	100
	Unsupport_reads-normalized	0.1	3.0	0.1	0	0	2.3	3.4
	Support_reads-normalized	99.9	97.0	99.9	100	100	97.7	96.6
	Proportion	0.999	0.970	0.999	1.000	1.000	0.977	0.966

Wastewater samples*	Analysis†	Mutations‡						
		C241T	C1059T	C3037T	C14408T	A23403G	G25563T	C28887T
	Mutation call	Present	Present	Present	Present	Present	Present	Present
COR-26TH-04-04-21-A	All_reads-actual	513	102	407	48	70	556	94
	Unsupport_reads-actual	0	0	0	0	0	91	57
	Support_reads-actual	513	102	407	48	70	465	37
	All_reads-normalized	100	100	100	48	70	100	94
	Unsupport_reads-normalized	0	0	0	0	0	16.4	57
	Support_reads-normalized	100	100	100	48	70	83.6	37
	Proportion	1.000	1.000	1.000	1.000	1.000	0.836	0.394
	Mutation call	Present	Present	Present	Present	Present	Present	Present
COR-27TH-04-04-21-A	All_reads-actual	358	6	23	25	9	33	31
	Unsupport_reads-actual	0	0	0	0	0	0	19
	Support_reads-actual	358	6	23	25	9	33	12
	All_reads-normalized	100	6	23	25	9	33	31
	Unsupport_reads-normalized	0	0	0	0	0	0	19
	Support_reads-normalized	100	6	23	25	9	33	12
	Proportion	1.000	1.000	1.000	1.000	1.000	1.000	0.387
	Mutation call	Present	Present	Present	Present	Present	Present	Present
DAL-Inf-04-19-21-A	All_reads-actual	25790	2420	5834	495	965	6530	1035
	Unsupport_reads-actual	10	568	4	0	3	2801	293
	Support_reads-actual	25780	1852	5830	495	962	3729	742
	All_reads-normalized	100	100	100	100	100	100	100
	Unsupport_reads-normalized	0.0	23.5	0.1	0	0.3	42.9	28.3
	Support_reads-normalized	100.0	76.5	99.9	100	99.7	57.1	71.7
	Proportion	1.000	0.765	0.999	1.000	0.997	0.571	0.717
	Mutation call	Present	Present	Present	Present	Present	Present	Present

* See Appendix Table 2

† Actual numbers of sequence reads spanning the mutation site (All_reads), the numbers of reads not supporting the presence of the mutation (Unsupport, the reference sequence), and the number of reads supporting the presence of the mutation (Support, mutant sequence) are shown. In addition, the normalized numbers of reads are shown. For normalization, read numbers greater than 100 were reduced proportionately to a total of 100 reads. For read numbers of 100 or less, the actual read number was retained. "Proportion" is the proportion of all reads that carry the mutation. Mutations were called present if the proportion was 0.05 or greater and the number of reads carrying the mutation was 2 or more. Otherwise, the mutation was called Absent. If the total number of reads was 5 or less, the call was "insufficient data" (Insuff).

‡ Mutations found in more than 20 variants including B.1.351.

Appendix Table 7. Detailed sequence read analysis of private mutations exhibited by B.1.351 individuals and found in wastewater samples in Linn County, Oregon and surrounding jurisdictions, March-April 2021

		Mutations‡														
Wastewater samples*	Analysis†	C355T	C904T	C2232T	G7393T	G8017T	C13059T	C15026T	G18382T	C18452T	C24579T	G25130T	A28254C	T28853G	G28048T§	A28111G§
ALB-Inf-03-26-21-A	All_reads-actual	67	43	61	5	119	54	15	34	29	10	13	27	16	48	11
	Unsupport_reads-actual	67	43	61	5	119	54	15	34	29	10	13	27	16	48	11
	Support_reads-actual	0	0	0	0	0	0	0	0	0	0	0	0	0	0	0
	All_reads-normalized	67	43	61	5	100	54	15	34	29	10	13	27	16	48	11
	Unsupport_reads-normalized	67	43	61	5	100	54	15	34	29	10	13	27	16	48	11
	Support_reads-normalized	0	0	0	0	0	0	0	0	0	0	0	0	0	0	0
	Proportion	0	0	0	0	0	0	0	0	0	0	0	0	0	0	0
	Mutation call	Absent	Absent	Absent	Insuff	Absent	Absent	Absent	Absent	Absent	Absent	Absent	Absent	Absent	Absent	Absent
ALB-Inf-03-31-21-A	All_reads-actual	57	40	45	2	74	73	11	75	75	26	28	71	37	108	32
	Unsupport_reads-actual	57	40	45	2	74	66	11	75	75	26	28	71	37	108	32
	Support_reads-actual	0	0	0	0	0	7	0	0	0	0	0	0	0	0	0
	All_reads-normalized	57	40	45	2	74	73	11	75	75	26	28	71	37	100	32
	Unsupport_reads-normalized	57	40	45	2	74	66	11	75	75	26	28	71	37	100	32
	Support_reads-normalized	0	0	0	0	0	7	0	0	0	0	0	0	0	0	0
	Proportion	0	0	0	0	0	0.096	0	0	0	0	0	0	0	0	0
	Mutation call	Absent	Absent	Absent	Insuff	Absent	Absent	Absent	Absent	Absent	Absent	Absent	Absent	Absent	Absent	Absent
ALB-Inf-04-07-21-A	All_reads-actual	682	540	139	12	209	527	24	152	110	31	64	172	119	486	109
	Unsupport_reads-actual	682	540	139	12	208	527	24	152	110	31	40	172	119	210	103
	Support_reads-actual	0	0	0	0	1	0	0	0	0	0	24	0	0	276	6
	All_reads-normalized	100	100	100	12	100	100	24	100	100	31	64	100	100	100	100
	Unsupport_reads-normalized	100	100	100	12	99.5	100	24	100	100	31	40	100	100	43.2	94.5
	Support_reads-normalized	0	0	0	0	0.5	0	0	0	0	0	24	0	0	56.8	5.5
	Proportion	0	0	0	0	0.005	0	0	0	0	0	0.375	0	0	0.568	0.055
	Mutation call	Absent	Absent	Absent	Absent	Absent	Absent	Absent	Absent	Absent	Absent	Present	Absent	Absent	Present	Present
ALB-Inf-04-14-21-A	All_reads-actual	6052	2796	730	797	6565	10121	2799	2534	4093	800	1000	649	1989	11410	2541
	Unsupport_reads-actual	6052	2796	730	797	6563	10121	2799	2534	4093	800	1000	649	1989	8	2
	Support_reads-actual	0	0	0	0	2	0	0	0	0	0	0	0	0	11402	2539
	All_reads-normalized	100	100	100	100	100	100	100	100	100	100	100	100	100	100	100
	Unsupport_reads-normalized	100	100	100	100	100	100	100	100	100	100	100	100	100	0.1	0.1
	Support_reads-normalized	0	0	0	0	0	0	0	0	0	0	0	0	0	99.9	99.9
	Proportion	0	0	0	0	0	0	0	0	0	0	0	0	0	0.999	0.999
	Mutation call	Absent	Absent	Absent	Absent	Absent	Absent	Absent	Absent	Absent	Absent	Absent	Absent	Absent	Present	Present
ALB-Inf-04-21-21-A	All_reads-actual	77	236	47	11	135	838	55	61	61	12	9	58	55	446	39
	Unsupport_reads-actual	77	236	47	11	135	838	55	61	61	12	5	58	55	23	10
	Support_reads-actual	0	0	0	0	0	0	0	0	0	0	4	0	0	423	29
	All_reads-normalized	77	100	47	11	100	100	55	61	61	12	9	58	55	100	39
	Unsupport_reads-normalized	77	100	47	11	100	100	55	61	61	12	5	58	55	5.2	10
	Support_reads-normalized	0	0	0	0	0	0	0	0	0	0	4	0	0	94.8	29
	Proportion	0	0	0	0	0	0	0	0	0	0	0.444	0	0	0.948	0.744
	Mutation call	Absent	Absent	Absent	Absent	Absent	Absent	Absent	Absent	Absent	Absent	Present	Absent	Absent	Present	Present
COR-25TH-04-04-21-A	All_reads-actual	1875	878	186	47	1312	1769	271	293	750	115	326	480	269	34	32
	Unsupport_reads-actual	1875	878	186	43	1312	1769	271	293	750	115	326	480	269	1	32
	Support_reads-actual	0	0	0	4	0	0	0	0	0	0	0	0	0	33	0
	All_reads-normalized	100	100	100	47	100	100	100	100	100	100	100	100	100	34	32
	Unsupport_reads-normalized	100	100	100	43	100	100	100	100	100	100	100	100	100	1	32
	Support_reads-normalized	0	0	0	4	0	0	0	0	0	0	0	0	0	33	0
	Proportion	0	0	0	0.085	0.000	0	0	0	0	0	0	0	0	0.971	0

Wastewater samples*	Analysis†	Mutations‡														
		C355T	C904T	C2232T	G7393T	G8017T	C13059T	C15026T	G18382T	C18452T	C24579T	G25130T	A28254C	T28853G	G28048T§	A28111G§
	Mutation call	Absent	Absent	Absent	Present	Absent	Absent	Absent	Absent	Absent	Absent	Absent	Absent	Absent	Present	Absent
COR-26TH-04-04-21-A	All_reads-actual	79	73	65	1	178	254	17	77	77	10	91	93	94	232	35
	Unsupport_reads-actual	79	73	65	1	178	254	17	77	77	10	91	93	94	70	27
	Support_reads-actual	0	0	0	0	0	0	0	0	0	0	0	0	0	162	8
	All_reads-normalized	79	73	65	1	100	100	17	77	77	10	91	93	94	100	35
	Unsupport_reads-normalized	79	73	65	1	100	100	17	77	77	10	91	93	94	30.2	27
	Support_reads-normalized	0	0	0	0	0	0	0	0	0	0	0	0	0	69.8	8
	Proportion	0	0	0	0	0	0	0	0	0	0	0	0	0	0.698	0.229
	Mutation call	Absent	Absent	Absent	Insuff	Absent	Absent	Absent	Absent	Absent	Absent	Absent	Absent	Absent	Present	Present
COR-27TH-04-04-21-A	All_reads-actual	98	20	46	0	115	249	0	57	31	30	7	58	32	85	38
	Unsupport_reads-actual	98	20	46	0	114	249	0	57	31	30	7	58	32	85	38
	Support_reads-actual	0	0	0	0	1	0	0	0	0	0	0	0	0	0	0
	All_reads-normalized	98	20	46	0	100	100	0	57	31	30	7	58	32	100	38
	Unsupport_reads-normalized	98	20	46	0	99.1	100	0	57	31	30	7	58	32	100	38
	Support_reads-normalized	0	0	0	0	0.9	0	0	0	0	0	0	0	0	0	0
	Proportion	0	0	0	0	0.009	0	0	0	0	0	0	0	0	0	0
	Mutation call	Absent	Absent	Absent	Insuff	Absent	Absent	Insuff	Absent	Absent	Absent	Absent	Absent	Absent	Absent	Absent
DAL-Inf-04-19-21-A	All_reads-actual	5517	2605	360	105	20000	3084	883	1324	1191	1379	648	1794	754	3541	638
	Unsupport_reads-actual	5517	2605	360	105	19999	3084	883	1324	1191	1379	648	1794	754	1531	163
	Support_reads-actual	0	0	0	0	1	0	0	0	0	0	0	0	0	2010	475
	All_reads-normalized	100	100	100	100	100	100	100	100	100	100	100	100	100	100	100
	Unsupport_reads-normalized	100	100	100	100	100	100	100	100	100	100	100	100	100	43.2	25.5
	Support_reads-normalized	0	0	0	0	0	0	0	0	0	0	0	0	0	56.8	74.5
	Proportion	0	0	0	0	0	0	0	0	0	0	0	0	0	0.568	0.745
	Mutation call	Absent	Absent	Absent	Absent	Absent	Absent	Absent	Absent	Absent	Absent	Absent	Absent	Absent	Present	Present

* See Appendix Table 2

† Actual numbers of sequence reads spanning the mutation site (All_reads), the numbers of reads not supporting the presence of the mutation (Unsupport, the reference sequence), and the number of reads supporting the presence of the mutation (Support, mutant sequence) are shown. In addition, the normalized numbers of reads are shown. For normalization, read numbers greater than 100 were reduced proportionately to a total of 100 reads. For read numbers of 100 or less, the actual read number was retained. "Proportion" is the proportion of all reads that carry the mutation. Mutations were called present if the proportion was 0.05 or greater and the number of reads carrying the mutation was 2 or more. Otherwise, the mutation was called Absent. If the total number of reads was 5 or less, the call was "insufficient data" (Insuff).

‡ Private mutations, namely mutations found in individual clinical B.1.351 clade 1 sequences that were not shared extensively by numerous other B.1.351 clade 1 sequences. Private mutations can be used for phylogenetic analysis and can occasionally be used to identify samples with a very recent common origin. See Appendix Table 9 for a list of private mutations exhibited by the Oregon B.1.351 individual clinical specimens.

§ These two private mutations are shared with B.1.1.7.

Appendix Table 8. Mutations present in B.1.351 shared with other variants

Mutation	Variants sharing the mutation
A10323G	B.1.404, B.1.582
11288Δ9	B.1.1.7, B.1.526, P.1
A22206G	B.1.526
G23012A	B.1.1.316, B.1.526, P.1
A23063T	B.1.1.7, P.1
C23664T	B.1.526

Appendix Table 9. Private mutations exhibited by B.1.351 cases in Linn County, Oregon, March—May 2021

GISAID accession number*	Private mutations†
EPI_ISL_2382524	G25130T, G7393T
EPI_ISL_1736532	G25130T, G18382T
EPI_ISL_1736521	C24579T
EPI_ISL_2139636	C24579T, C2232T
EPI_ISL_2382527	C24579T, C904T, C18452T, T28853G
EPI_ISL_2202145	G8017T
EPI_ISL_2139637	C355T
EPI_ISL_2139638	C355T
EPI_ISL_2139644	C355T
EPI_ISL_2139639	G29254T
EPI_ISL_2339336	G29254T
EPI_ISL_2086679	G29254T
EPI_ISL_2086678	none
EPI_ISL_2086694	none
EPI_ISL_1964160	A28254C
EPI_ISL_2250177	A28254C, C15026T
EPI_ISL_1866415	G29402T
EPI_ISL_1999265	C13059T, G28048T, A28111G
EPI_ISL_1737841	C11514T

* GISAID accession numbers are listed for each specimen. The sequences are listed in the same order, top to bottom, in which they appear in Figure 1 and Appendix Figure 2.

† Private mutations are defined as mutations that occur in small numbers of sequences and that do not define a variant, clade or sub-clade.

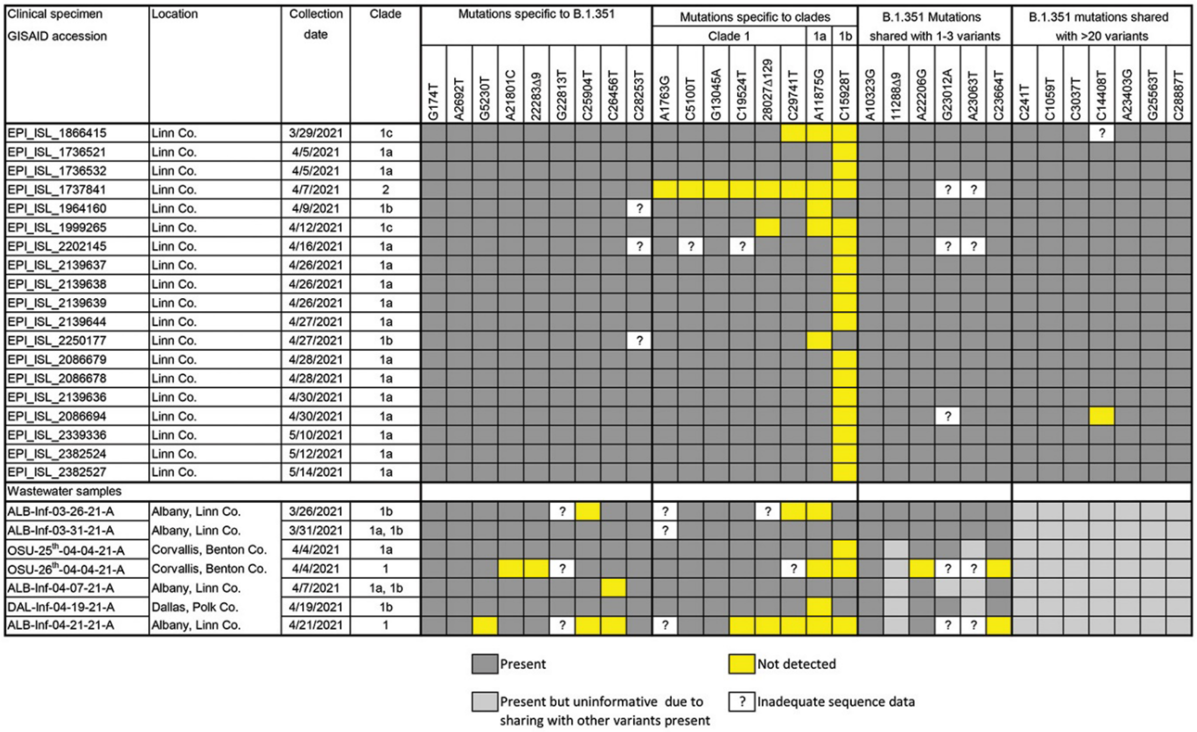

Appendix Figure. Mutations detected in B.1.351 clinical specimens and wastewater samples in Linn County, Oregon and surrounding jurisdictions, March-May 2021. Clinical specimens are identified by their GISAID accession numbers. Wastewater samples are identified by their field collection identifier. Cities where individual clinical specimens were collected are not provided to reduce identifiability of cases. Mutations are described relative to the Wuhan-Hu-1 reference sequence. Mutations were considered present in wastewater samples if at least 5% of sequence reads exhibited the mutation and at least two reads exhibited the mutation. Inadequate sequence data was defined as a gap spanning a mutation in individual specimen data or fewer than six reads spanning a mutation in the case of wastewater sample data. Mutations were considered uninformative when other variants with shared mutations were present. Additional mutations found in small numbers of individual clinical specimens (private mutations) are listed in Appendix Table 9.
